# Investigating Preservice Teachers' Competence to Notice Ethnic Microaggressions in the Classroom

**DOI:** 10.1002/jcop.70056

**Published:** 2025-11-19

**Authors:** Sauro Civitillo, Sabine Glock, Luca Alexander Naudszus, Louisa Venhoff, Dwayne Lieck, Philipp Jugert

**Affiliations:** ^1^ Utrecht University Utrecht The Netherlands; ^2^ University of Wuppertal Wuppertal Germany; ^3^ ETH Zürich Zürich Switzerland; ^4^ University of Vienna Vienna Austria; ^5^ University of Freiburg Freiburg im Breisgau Germany; ^6^ University of Duisburg‐Essen Duisburg and Essen Germany

**Keywords:** Germany, microaggressions, preservice teachers, racism, teacher noticing

## Abstract

Ethnic microaggressions convey insensitivity or demean a person's ethnic identity and have been documented in educational settings targeting minoritized students. Drawing on the racial noticing framework and insights from teacher psychological characteristics, the present study examined preservice teachers' competence to *attend* and *interpret* ethnic microaggressions and explored the attitudinal dispositions (i.e., implicit and explicit prejudice, critical reflection, and empathy) that contribute to teachers' awareness of these discriminatory actions. Using a vignette design across two experimental studies in Germany (Study 1, *N* = 147, M_age_ = 23.7; 88% females; Study 2, *N* = 184, M_age_ = 23.2; 64.7% females), our results indicated that participants demonstrated noticing ability in detecting microaggressions targeting Turkish heritage students. We found less consistent evidence for the role of participants' attitudinal dispositions as predictors of racial noticing. In Study 1, implicit prejudice was negatively related to attending microaggressions, while, surprisingly, explicit prejudice was positively associated with interpreting microaggressions. However, these findings did not replicate in Study 2. Additionally, higher levels of critical reflection were positively linked to a greater ability in attending and interpreting ethnic microaggressions. We discuss implications for teacher education on noticing ethnic microaggressions in schools.

Ethnic microaggressions are a complex and multifaceted phenomenon that manifests in both overt and subtle interpersonal ways during everyday exchanges and communicates bias toward persons of racially and ethnically minoritized groups (Torino et al. 2018). Overt microaggressions (i.e., microassaults) involve the blatant differential treatment of ethnic minorities, based on certain personal characteristics or group affiliations that are perceived as inferior to those of the dominant group (Torino et al. 2018). Subtle forms of microaggressions (i.e., microinsults or microinvalidations) occur through behaviors or verbal comments that convey insensitivity or demean a person's ethnic identity (Sue et al. 2007). These exchanges are brief, common, and ambiguous and thus “often dismissed and glossed over as being innocent and innocuous” (Sue et al. 2007, 273). However, recent systematic reviews and meta‐analyses have shown that experiences of microinsults and invalidations are associated with negative adjustment to a similar degree as microassaults (Civitillo et al. 2024; P. L. Costa, McDuffie, et al. 2023; Lui and Quezada 2019).

In the current study, we drew on the recently introduced framework of racial noticing (Shah and Coles 2020), which makes the facets of racism in schools visible, to investigate whether preservice teachers in Germany distinguish ethnic microaggressions in teacher−student interactions by systematically varying the level of ambiguity of the deliverer's intentions. Using an experimental design, we presented participants with written vignettes of ethnic microaggressions and examined whether participants perceived the deliverers of microaggressions as biased (i.e., perceived microaggressions) and the potential psychological harm of such exchanges (i.e., perceptions of students' negative sense of school belonging). We chose to present interactions involving Turkish heritage students because they represent the biggest ethnic minority in Germany and are often exposed to high levels of interpersonal discrimination in schools (Vietze et al. 2023). Turkish heritage students, in particular boys, are often portrayed through deficit narratives that label them as underachievers in public and academic discourses (Çelik 2015). Despite a growing acknowledgment of the existence of racism in public debates and German schools, whether a situation is judged to be racist also depends on the target group. While anti‐Black racism and antisemitism are frequently identified, discrimination against Turkish minorities has received less attention (DeZIM [Deutsches Zentrum für Integrations‐ und Migrationsforschung] 2022).

Moreover, building on the growing body of literature identifying specific aspects of teacher psychology that contribute to inequitable treatment of ethnically minoritized students (Denessen et al. 2022; Turetsky et al. 2021), our study further explored whether preservice teachers' attitudinal dispositions can account for these differences in noticing microaggressions. Notably, racial noticing explicitly acknowledges the role of teachers' attitudinal dispositions, such as the rejection of prejudicial views about minoritized groups of students. Differences in teacher noticing are likely shaped not only by negative views but positive teachers' dispositions such as critical reflection (i.e., an individual's awareness of oppressive systemic forces in society; Heberle et al. 2020) and empathy (i.e., the capacity to share the emotional state of another, and identify with other, adopting their perspective; De Waal 2008) might play an important role as well. Accordingly, in Study 1, we investigated negative dispositions, specifically, implicit and explicit prejudice. In Study 2, besides prejudice, we also examined positive attitudinal dispositions, namely critical reflection and empathy. We pursued this approach because teachers, as all members of society, may display negative biases of marginalized groups, but at the same time, culturally responsive teachers are those who promote critical consciousness and show empathy toward their students (Gay 2018; Ladson‐Billings 1995; Warren 2018). Thus, when preparing teachers, training targeting both negative and positive dispositions might be a more effective means to dismantle racism and oppressions in schools than targeting either in isolation. We focus on preservice teachers, who may struggle to recognize and interpret ambiguous discriminatory interactions in the classroom because they are still learning the fundamentals of the profession, yet are later encouraged to serve as antiracist educators.

## 1 Teacher Noticing of Ethnic Microaggressions

Following the taxonomy of Sue et al. (2007), in the current study, we examined scenarios depicting microinsults and microinvalidations. Microinsults are actions or remarks that convey rudeness, insensitivity, or diminish a person's ethnic identity. This could occur when a teacher expresses surprise upon learning that a student from a marginalized background reports good grades. The underlying message is that such students are not expected to excel academically. Microinvalidations are behaviors or verbal comments that exclude, negate, or dismiss the psychological experiences, thoughts, or feelings of the target group, for example, when a teacher assumes that all Turkish heritage students behave in the same way. The underlying message of such interactions is that there is no variation within this group.

In the school ecology, research consistently highlights the frequent occurrence of microassaults, such as name‐calling and physical aggressions (English et al. 2020; Bayram Özdemir et al. 2019). At the same time, a study by Juang et al. (2021) illustrates the widespread occurrence of foreign objectification, a form of microinsult. Among more than 800 ethnically minoritized secondary school students in Germany, two‐third of the participants reported being asked “where are you really from?” because of their cultural background/appearance, indicating that others had commented on or were surprised by how well they spoke German, and that about one‐third had been spoken to in an unnecessarily slow or loud way (Juang et al. 2021). In three qualitative studies with ethnically diverse samples of emerging adults (Colak et al. 2020; Kehl et al. 2024; Moffitt et al. 2019), teachers, rather than peers or classmates, were frequently recalled as the deliverers of ethnic microaggressions. In another qualitative study (Artamonova 2018), students in a German multi‐ethnic vocational school reported that microaggressions were used by the teacher to create a “playful” atmosphere in the classroom, capitalizing on ambiguity or interpretational vagueness. Because of the ambiguous nature of ethnic microaggressions in teaching and learning situations, it is important to explore the factors that contribute to teachers' awareness of these discriminatory actions, particularly when these exchanges occur between teachers and students (Fu et al. 2024; Steketee et al. 2021).

It is relevant for prospective teachers to become aware of and understand the implications of subtle forms of racism, essentially developing the ability to notice ethnic microaggressions. While in everyday conversation the term noticing simply means the act of observing or recognizing something, teacher noticing is an established theoretical framework developed in the field of mathematics education that refers to the construction of meaning about what teachers observe in the classroom (van Es and Sherin 2008; Weyers et al. 2024). Shah and Coles (2020) expanded the literature on teacher noticing to teachers' attention and sense‐making of ethnic and cultural aspects of teaching and learning. Importantly, racial noticing goes beyond a mere cognitive ability; it is grounded in the assumption that power in an unequal and stratified society is also reflected in classroom activities.

Shah and Coles (2020) describe three interrelated, sequential processes of racial noticing: *attending*, *interpreting*, and *responding*. Attending refers to becoming aware of a given phenomenon, interpreting refers to understanding the implications of that phenomenon, and responding refers to planning instruction as a result of having attended to and interpreted a classroom phenomenon. Although responding is crucial, our interest in the current study is in attending and interpreting because without mastering these two preliminary steps, responding is unlikely to be effective. Indeed, prior research has shown that teachers may not have received enough training to attend to microaggressions because they may lack the knowledge to distinguish racist comments such as microaggressions from other forms of inappropriate behavior (Pearce 2019). In semi‐structured interviews with in‐service teachers, Burleigh and Wilson (2021) found that microaggressions were not brought to the teachers' attention during their professional training. As a result, many teachers felt ill‐equipped to handle microaggressions and relied solely on previous anti‐bullying training or classroom management and discipline issues. Furthermore, when the perpetrator of a microaggression is an authority figure such as a teacher, it may be challenging to link these incidents to racism because teachers tend to position themselves as “good teachers,” resisting an awareness of potentially acting racist themselves (Hölscher et al. 2024; Kehl et al. 2024; Legette et al. 2023).

Although ethnic microaggressions are microlevel social practices in the form of commonplace everyday indignities (Sue et al. 2007), it is crucial for teachers to understand such experiences within the macrosystem of racial politics, structural racism, and historical oppression that shape society (Rogers et al. 2021). Recognizing the broader contextual nature of individual experiences and structural systems allows to recognize that microaggressions are not merely interpersonal interactions, but rather a product of broader systemic inequities and historical legacies of oppression (Syed 2021). While the experiential reality of the target is fundamental to microaggression theory (Sue 2017), teachers must skillfully attend to how students may be exposed to these subtle forms of racism in classroom activities and interactions, yet they often feel ill‐equipped to address them (OECD 2019). Thus, we investigated the extent to which preservice teachers attend to the nuances of the ambiguity inherent to ethnic microaggressions, independently of the target's self‐reported experience.

Becoming aware of an interpersonal racist exchange represents an important first step, but it does not necessarily mean that teachers fully understand and interpret the deeper implications of such encounters for ethnically minoritized students (Shah and Coles 2020). Microaggressions may, in fact, appear harmless, leading to their dismissal as a micro form of racism (Kohli et al. 2017). Well‐intended individuals might downplay the severity of ethnic microaggressions, attributing them to the perceived oversensitivity of the target (West 2019). However, research with youth and adolescents has consistently shown that microaggressions reinforce exclusionary messages about who belongs and who does not. Within the context of students' teacher or peer relationships, experiencing microaggressions can severely damage students' sense of school belonging (D'Hondt et al. 2015; Guerra et al. 2019; Montoro et al. 2021). School belonging refers to students perceived psychological membership in the school environment (Goodenow 1993). Students who report higher levels of school belonging tend to score higher on standardized tests, hold more favorable attitudes toward school, and are less likely to drop out of school (Allen et al. 2018). Importantly, the consequences of experiencing racism on student' sense of belonging may vary when teachers (vs. peers) engage in such actions. Montoro et al. (2021) found among Black, Asian American, and Latinx youth that experiences of racism from adults at school are more likely to report lower school belonging, whereas perceived discrimination from peers was not associated directly with adolescents' sense of belonging. Hence, it is important to consider whether teachers can grasp the potentially damaging consequences of experiencing microaggressions on students' sense of school belonging. We therefore examined preservice teachers' perceptions of students' negative sense of school belonging, particularly in circumstances where the deliverers of microaggressions are teachers. In what follows, we review the literature on teacher psychological characteristics, highlighting the specific ways they might contribute to understanding their relation to teacher noticing of ethnic microaggressions.

## 2 Implicit and Explicit Prejudice of Teachers

Although both implicit and explicit prejudice develop from repeated exposure to pairings of a social group with a particular characteristic, theoretically, they are distinct constructs (Stark et al. 2023). According to the associative‐propositional evaluation model (Gawronski and Bodenhausen 2006), implicit prejudice reflects automatic affective reactions resulting from the particular associations that are automatically activated when an individual encounters a relevant stimulus. Explicit prejudice should be conceived as positive and negative attributes that individuals believe members of a social group to possess, including behavioral tendencies, values, personality traits, and preferences (Stark et al. 2023). Among teachers, research has illustrated that implicit attitudes toward ethnically minoritized students are generally negative (S. Costa, Pirchio, et al. 2023; Glock and Kleen 2023; van den Bergh et al. 2010), whereas explicit attitudes are either neutral or positive (S. Costa, Pirchio, et al. 2023; Hachfeld et al. 2011; Vezzali et al. 2012). Importantly, there is mixed evidence regarding the associations and the predicting validity of implicit and explicit attitudes with teachers' stereotypical expectations and discriminatory behavior. On the one hand, both in‐service and preservice teachers with more negative implicit attitudes reported a lower preference to teach ethnically minoritized students, whereas explicit attitudes were not a significant predictor of this judgment (Glock and Böhmer 2018). On the other hand, Kumar et al. (2015) found that implicit and explicit attitudes among White teachers related differently to classroom instructions. Implicitly favorable attitudes toward White relative to minoritized students were associated with lower scores on promotion of respect in the classroom, and explicit negative attitudes toward minoritized and students from lower socioeconomic status were related to performance‐focused instructional practices (Kumar et al. 2015). This suggests that both implicit and explicit prejudice are worth investigating because they may be differently associated with discriminatory behaviors.

Implicit attitudes might be the result of widespread socialization within the family, but also school, and might create strong associations in the long‐term memories of substantial groups of people (Payne et al. 2017), including teachers (Starck et al. 2020). Because of the unconscious influences underlying prejudiced attitudes, individuals may engage in microaggressions without intention and awareness and may be motivated to deny evidence of the power‐based nature of their action (Dovidio et al. 2018). Unlike overt racism, which most people are willing to acknowledge as harmful, microaggressions are typically considered as small and innocuous acts (Sue 2010). Individuals high in implicit prejudice are likely to seek nonracial explanations for their behavior to preserve a non‐prejudiced self‐image (Dovidio et al. 2017). For example, across two well‐powered studies with in‐service teachers, Tropp and Rucinski (2022) found that teachers with higher implicit pro‐White/anti‐Black biases reported lower intentions to engage in race‐related discussions with students, controlling for participants' self‐reported internal and external motives to be unbiased. Yet, to our knowledge, there are no studies that have specifically investigated the relation between implicit prejudice and perceptions of ethnic microaggressions. Microaggressions may go unnoticed by deliverers because they often occur as an automatic reaction to unconscious biases and stereotypes (Steketee et al. 2021). Therefore, we theorized that high levels of implicit prejudice may also reduce participants' perceptions that more subtle forms of racism, such as microaggressions, constitute bias and that the consequences are significant.

We also investigated explicit attitudes by examining subtle prejudice and color‐evasiveness. Following Pettigrew and Meertens (1995), subtle prejudice takes the form of exaggeration of cultural differences between groups and denial of positive emotions. The former reflects beliefs about perceived dissimilarity with the out‐group, considered a foundation form of prejudice, while the latter captures emotional reactions to out‐groups and may act as a more covert indicator of subtle prejudice. Among highly educated, younger, and more progressive individuals, subtle prejudice predicts out‐group rejection in a similar way to blatant prejudice (Meertens and Pettigrew 1997). Yet subtle prejudice is relatively more socially accepted and seen as less discriminatory (Rattazzi and Volpato 2003; Stanke et al. 2024) because more progressive individuals generally reject overt, blatant expressions of intergroup hostility.

Besides subtle prejudice, color‐evasiveness, often considered a form of microaggression in itself (Sue et al. 2007) and associated with negative intergroup attitudes and behavior (Yi et al. 2023), aims at stressing individuality and uniqueness and ignoring ethnic group differences. In doing so, this ideology tends to downplay the existence of racism and its consequences. Research from the US among White undergraduates (Mekawi et al. 2017; Mekawi and Todd 2021) found a positive link between color‐evasion and acceptability of ethnic microaggressions. Specifically, individuals who endorse high levels of color‐evasiveness tend to perceive the commission of microaggressions as acceptable, but do not necessarily agree with the content of the microaggressions per se. Previous work with teachers in Europe (Hagenaars et al. 2023), including Germany (Schotte et al. 2022), has shown that teachers are likely to endorse high levels of color‐evasiveness. Yet, prior research with preservice teachers in Germany (Civitillo et al. 2021) found that color‐evasiveness consists of two distinct factors, stressing similarities (i.e., emphasizing common ground among heterogeneous groups of students) and ignoring differences (e.g., directing attention to the individual, and not to social group membership). While the first dimension is endorsed more highly among preservice teachers in Germany, the second dimension more clearly taps into classic operationalizations of color‐evasiveness in the US context where this seems to be the dominating ideology among pre‐ and in‐service teachers (Cadenas et al. 2021; DeCuir‐Gunby et al. 2020). In prior work, only ignoring differences, but not stressing similarities, was negatively associated with multiculturalism and polyculturalism and positively associated with cultural‐diversity related stress (Civitillo et al. 2021). It is likely that preservice teachers who ignore social group membership in encounters between individuals are less able to attend to ethnic microaggressions and to understand their implications because accounting for group membership is a necessary first step to comprehending the reason why microaggressions may be hurtful to ethnically minoritized members. Thus, in addition to subtle prejudice, we investigated how the ignoring differences dimension of color‐evasiveness is associated with the recognition and interpretation of microaggressions in the classroom.

## 3 Critical Reflection and Empathy

Critical consciousness reflects an individual's awareness of oppressive systemic forces in society and may translate into a sense of efficacy to work against oppression (Heberle et al. 2020). Critical consciousness is a core of social justice teaching and reflects a heightened awareness of the positioning of social groups (including ethnically minoritized groups) in society and the power structures that shape these positionings (Freire 1978). In modern conceptualization, critical consciousness is typically thought to consist of three dimensions: awareness of oppressive systems (critical reflection), a sense of efficacy (critical motivation), and active engagement (critical action) against oppression (Schwarzenthal et al. 2022). This study focuses on critical reflection, because this dimension of critical consciousness is considered foundational for fostering critical action, with reflection and action reinforcing each other in a continuous cycle (Freire 1978). Previous studies have shown that in classrooms, in which teachers promote critical reflection, students feel empowered to talk about the roots and implications of social inequity and systemic racism (Byrd 2017; Schachner et al. 2021). While originally conceptualized solely from the perspective of socially oppressed groups as a means of empowerment, members of socially dominant groups can also develop critical reflection. However, while the literature on the development of youths' critical reflection has rapidly increased in the last decade (Diemer et al. 2021), research among teachers remains limited. During teacher education, it is convincible that preservice teachers may increase their critical awareness and supporting their engagement in culturally responsive practices (Civitillo et al. 2018; European Commission 2017). Ethnic microaggressions can be seen as examples of the influence of systems of oppression in everyday encounters. Thus, individuals high in critical reflection should be more attuned to recognize such actions as problematic.

Empathy reflects the ability to understand another's perspective in combination with emotional engagement (De Waal 2008). Empathy is an important psychological characteristic which facilitates culturally responsive teaching because it allows teachers to better understand the students they teach (Warren 2018). More specifically, empathy permits teachers to critique and elaborate on existing knowledge and acquire new knowledge of students' personal and social situations (Civitillo and Juang 2020). If teachers do not empathize or understand the disparities faced by ethnically minoritized students (e.g., systemic racism, institutional discrimination), they may be lacking awareness and concern for the consequences of discriminatory actions. Teacher noticing, which might be resembling as an act of perspective taking, requires engaging in the processes of attending and interpreting, before making subsequent professional decisions. Thus, we explored empathy to strengthen the links with teacher noticing.

## 4 Current Study

We preregistered our hypotheses, study design, and sampling strategy before data collection on www.aspredicted.org (Study 1: https://aspredicted.org/8dgd-yt8d.pdf; Study 2: https://aspredicted.org/srwp-j37t.pdf). Across both studies, we tested the following predictions: Participants' ratings of perceived microaggression (H1a) and projected negative effects on student sense of school belonging (H1b) would increase across each of the conditions of microaggression ambiguity (i.e., control, high ambiguity, and low ambiguity). In Study 1, we hypothesized that both explicit measures (i.e., color‐evasiveness and subtle prejudice toward individuals of Turkish origin) and implicit measures (greater negative implicit attitudes toward Turks) would be associated with lower ratings of perceived microaggression (H2a) and lower ratings of projected negative effects on student sense of school belonging (H2b) across the microaggression ambiguity conditions of two sets of vignettes. In Study 2, we replicated the predictions from H1a to H2b, but additionally, we hypothesized that participants who report high levels of critical reflection and empathy would report higher ratings of perceived microaggression (H3a) and higher ratings of projected negative consequences on students' sense of school belonging (H3b) across the microaggression ambiguity conditions. We controlled for gender and migration background (operationalized here as being born abroad or having at least one parent born outside of Germany) because these variables have been shown to relate to levels of prejudice among preservice teachers (e.g., Gegenfurtner 2021). Additionally, because prior research indicates that practical teaching experience, which preservice teachers in Germany typically gain through mandatory internships, explains more variance in teaching skills than personality traits (e.g., Biermann et al. 2015), we also controlled for this variable.

## 5 Study 1

### 5.1 Methods

#### 5.1.1 Participants

We determined the required sample size by an a priori power analysis using G*Power 3.1 (Faul et al. 2009). For mixed ANOVA (H1a and H1b), with three levels factor for vignette condition (i.e., control, high ambiguity, and low ambiguity), two levels for the between‐subject factor for type of scenario (school performance vs. school normative behavior), *α* set at 0.05, a power of 0.95, the analysis indicated a minimum sample size of 142 participants. In addition, due to limited study resources (each participant received a 5‐Euro gift card), a stopping rule of 150 participants was applied. The sample included 150 preservice teachers who studied in the Ruhr Area, Germany. Three participants reported to have a Turkish heritage and thus were excluded from later analyses, because their inclusion could have influenced the results given the focus on ethnic microaggressions toward this group. Of the remaining 147 respondents (M_age_ = 23.7 years old, SD = 5.4; 88% females), less than 5% had an immigrant background. Participants were either enrolled in a primary (17%) or secondary (83%) teacher education program.

#### 5.1.2 Measures

##### 5.1.2.1 Implicit Prejudice

To measure implicit prejudice, we employed a Single Attribute Implicit Association Test (SA‐IAT) (Greenwald et al. 1998; Penke et al. 2006). Six common German male first names (Tim, Paul, Jonas, Niklas, Lukas, and Finn) and six Turkish male first names (Yusuf, Caner, Oktay, Mehmet, Mustafa, and Muhammed) indicated the categories ethnic minority and ethnic majority. We used six negative attributes (i.e., intolerant, segregated, nonconformist, religious, traditional, misogynistic) derived from previous research as stimuli for the second target attribute category. We were interested in whether they are more strongly associated with Turkish when compared to German names on an implicit level. A *D* score to quantify the strength of this difference in association was calculated for each individual following the Improved Scoring Algorithm (Greenwald et al. 2003) by subtracting average response times for the consistent trials from the average response of inconsistent trials and then dividing the result by the standard deviation of all trials of this individual. Consistency refers to the consistency of a trial with the presence of an implicit prejudice. A consistent trial includes the pairing of ethnic minority names with negative words, whereas an inconsistent pairing is the pairing of ethnic majority names with negative words, so that a positive *D* score indicates a stronger implicit prejudice. We calculated the *D* score as recommended by Greenwald et al. (2003), excluding trials with latencies over 10,000 ms and controlling for unfaithful test execution. Split‐half reliability assessed through the odd‐even method was sufficient (*r* = 0.84).

##### 5.1.2.2 Vignettes

Participants were presented with two sets of scenarios depicting interactions between a teacher and a student of Turkish origin, signaled by the students' names (e.g., Emre, Serkan), at a fictitious school (see Supporting Information Materials for the full description). To reduce possible confounding variables like student gender, all vignettes pertained to a male student. One set of vignettes focused on school performance, where teachers interacted with students regarding their academic or personal abilities. The second set presented normative behavior situations, where teachers addressed expectations or judgments based on societal norms, particularly those related to culture and gender. Each set included three conditions (i.e., control, high ambiguity, and low ambiguity). These six vignettes were selected from an original set of 11 vignettes developed by the authors after reviewing the microaggression literature (Artamonova 2018; Colak et al. 2020; Juang et al. 2021; Moffitt et al. 2019) and then refined through pilot testing with different preservice teachers (*N* = 25) at the university where the study was conducted.

##### 5.1.2.3 Perceived Microaggressions

Adapted from previous research on microaggressions (Basford et al. 2014; Tao et al. 2017), perceived microaggression was measured on a three‐item scale (e.g., “The teacher was insensitive about the student's cultural group”) rated on a Likert scale from 1 (*strongly disagree*) to 7 (*strongly agree*). Higher scores indicated high levels of perceived microaggressions. The scale demonstrated adequate reliability across all vignettes (for school performance: *ω* = 0.77 in the control condition, *ω* = 0.83 in the high ambiguity condition, and *ω* = 0.45 in the low ambiguity condition; for school normative behavior: *ω* = 0.84 in the control condition; *ω* = 0.86 in the high ambiguity condition, and *ω* = 0.72 in the low ambiguity condition). The standardized factor loadings for these items were good (for school performance: from 0.59 to 0.83 in the control condition, from 0.70 to 0.84 in the high ambiguity condition, and from 0.19 to 0.74 in the low ambiguity condition; for school normative behavior: from 0.68 to 0.86 in the control condition; from 0.76 to 0.88 in the high ambiguity condition, and from 0.51 to 0.90 in the low ambiguity condition).

##### 5.1.2.4 Students' Negative Sense of School Belonging

Students' negative sense of school belonging was measured on a three‐item scale developed to reflect the degree to which participants perceived that the ethnic microaggression perpetuated by the teacher would lower the student's sense of school belonging (adapted from Offermann et al. 2013, e.g., “The teacher's answer has an impact on [student's name] sense of belonging to the class.” Participants responded on a Likert scale, ranging from 1 (*strongly disagree*) to 7 (*strongly agree*). Higher scores indicated high levels of students' negative sense of school belonging. The scale demonstrated adequate reliability across all vignettes (school performance: no microaggression *ω* = 0.61, high ambiguity *ω* = 0.77, and low ambiguity *ω* = 0.67; school normative behavior: no microaggression *ω* = 0.67; high ambiguity *ω* = 0.82, and low ambiguity *ω* = 0.78). The standardized factor loadings for these items were suboptimal. For school performance, loadings ranged from 0.26 to 0.39 in the control condition (with one item producing a Heywood case *λ* = 1.17); from 0.46 to 0.53 in the high ambiguity condition (with one Heywood case, *λ* = 1.08), and from 0.34 to 0.36 in the low ambiguity condition (with one Heywood case *λ* = 1.21). For school normative behavior, loadings ranged from 0.34 to 0.97 in the control condition, were 0.51 for two items in the high ambiguity condition (with one Heywood case, *λ* = 1.11), and ranged from 0.50 to 0.65 in the low ambiguity condition (with one Heywood case, *λ* = 1.07). These lower loadings and occasional Heywood cases likely reflect the experimental sensitivity of the measures, which can produce variability across conditions. Nevertheless, because reliabilities were adequate for almost all scales, we retained all items for subsequent analyses.

##### 5.1.2.5 Subtle Prejudice Toward Turkish Origin Individuals

We measured subtle prejudice by using a German adaptation (Ganter 2001) of the Subtle Prejudice Scale by Pettigrew and Meertens (1995). This scale consisted of two subscales: (1) the exaggeration of cultural differences (three items; e.g., “Turkish origin individuals living here teach their children values and skills different from those required to be successful in Germany”; “Turkish origin individuals are different from Germans in the values that they teach their children”; *ω* = 0.72) rated on a scale from 1 (*very great similarities*) to 7 (*very great differences*), and (2) the negation of positive emotions (two items; e.g., “How often have you felt sympathy for Turkish origin individuals”; *r*_Spearman‐Brown_ = 0.68, *p* < 0.001), rated on a scale from 1 (*very often*) to 7 (*very seldom*). Higher scores indicated high levels of subtle prejudice. The fit of the two‐factor model was good *χ* ^2^(4) = 9.274, CFI = 0.974, RMSEA = 0.095 [90% CI 0.000; 0.177], and SRMR = 0.051. The standardized factor loadings for these items ranged from 0.54 to 0.89.

##### 5.1.2.6 Color‐Evasiveness

Color‐evasiveness consisted of four items tapping into the ignoring differences dimension (Civitillo et al. 2021; e.g., “Cultural background does not play a role in how students are treated”; *ω* = 0.66). Responses were given on a 7‐point scale ranging from 1 (*strongly disagree*) to 7 (*strongly agree*). The fit of the one‐factor model was good *χ* ^2^(2) = 2.245, CFI = 0.997, RMSEA = 0.029 [90% CI 0.000; 0.170], and SRMR = 0.027. The standardized factor loadings for these items ranged from 0.23 to 0.89.

### 5.2 Study Design and Procedure

Using a between‐subject design, participants were randomly assigned one of the sets of scenarios containing the three vignettes. Before reading each vignette, participants completed the SA‐IAT to avoid carry‐over effects. After reading each vignette, participants responded to items on the perceived microaggression and student sense of school belonging scales. Subtle prejudice and color‐evasiveness were assessed subsequently, followed by demographics.

The experiment was conducted online. Participants were recruited through the university website, introductory courses, on campus, and on a Facebook page for preservice teachers attending teacher training in the Ruhr area. Data were collected in April through August 2020 online using Inquisit 5 and Unipark. Each participant consented to the use of their anonymized data and received a written debriefing explanation of the real goals at the end of the study. Ethical approval for this study was obtained from the Ethics Committee at the Institute of Psychology (University of Duisburg‐Essen).

Five of the authors are White German, while the first author is White Italian. While some of the authors have a family history of immigration, all were born and raised in Germany. The first author is a migrant, having resided in Germany for 8 years, and an extended period of time in the Netherlands. Notably, while none of the authors have experienced ethnic microaggressions, our research focuses on racism, discrimination, and stereotypes within educational contexts. Over the years, we have also engaged with preservice teachers, including those from ethnically minoritized backgrounds, in both our research and teaching. These interactions have been crucial to shaping our understanding of classroom dynamics and the subtle nature of ethnic microaggressions. In fact, the development of study materials (e.g., vignettes) was informed by classroom discussions and collaborative reflections with our students, who helped us grasp the nuanced forms of bias that occur in educational settings. These insights complemented the existing literature and helped us design more context‐sensitive scenarios. While we sought to ground our work in both empirical research and student‐informed perspectives, we recognize, however, that our positionalities may still limit the depth and sensitivity with which we interpret and represent the lived experiences of minoritized students. We also acknowledge the importance of continuing consultation and coproduction with members of the communities most affected by ethnic microaggressions.

### 5.3 Analytic Strategy

We first identified univariate outliers by using the *z*‐score transformation, which computes the distance in standard deviation from the mean. We checked whether the data violate the assumption of sphericity using Mauchly's test. To examine H1a and H1b, we conducted a 2 × 3 ANOVA for each dependent variable separately (i.e., perceived microaggressions and projected negative student sense of school belonging), with type of student behavior (i.e., school related performance vs. school normative behavior) as between‐subject factor, and microaggression ambiguity condition (i.e., control, high ambiguity, and low ambiguity) as the repeated measure factor. To test H2a and H2b, we ran separate multiple regression analyses regressing perceived microaggressions and projected negative student sense of school belonging on color‐evasiveness beliefs, subtle prejudice toward Turkish origin individuals, and implicit ethnic prejudice, while controlling for participants' migration background, weeks of teaching experience, and gender. The two dependent variables, perceived microaggressions and projected negative student sense of school belonging, were calculated as a mean score for each participant across the microaggression ambiguity conditions.

For the SA‐IAT, in line with well‐established Greenwald et al. recommendations (2003), we excluded participants who reported more than 10% trials below 300 ms; more than 10% trials above 10,000 ms; and more than 25% missing trials. Based on these recommendations, none of the participants were excluded. SPSS for Windows v29 was used for data processing and hypotheses testing. *R* was used for CFAs and calculating *D* scores of the SA‐IAT. Missing values on the main variables were less than 1%.

### 5.4 Results

Table 1 shows descriptive statistics and bivariate correlations for all key variables. There was only a positive correlation between the exaggeration of cultural differences and the negation of positive emotions (*r* = 0.40, *p* < 0.01). Mauchly's test indicated that the assumption of sphericity had been violated *χ* ^2^(2) = 0.950, *p* < 0.05. Thus, degrees of freedom were corrected using Huynh−Feldt estimate of sphericity (*ε* = 0.70) when reporting results for H1a. For H2b, Mauchly's test indicated that the assumption of sphericity held, *χ* ^2^(2) = 0.966, *p* = 0.087. Microaggression ambiguity had a significant main effect on perceived microaggressions, *F*(2, 143) = 338.02, *p* < 0.001, *η*_p_^2^ = 0.825, supporting H1a. In the school performance vignettes, participants perceived significantly more microaggressions in the low‐ambiguity condition (M = 5.87, SD = 0.86) than in the high‐ambiguity condition (M = 4.57, SD = 1.32), *t*(72) = −8.137, *p* < 0.001, *d* = 1.46.^1^ Both were significantly higher than the control condition (M = 2.08, SD = 1.09), all *ps* < 0.001. In the school normative behavior vignettes, the same pattern was observed: low‐ambiguity condition (M = 5.56, SD = 1.21) was higher than high‐ambiguity condition (M = 4.45, SD = 1.39), *t*(73) = −6.93, *p* < 0.001, *d* = 1.89, and control condition (M = 2.96, SD = 1.35), *t*(73) = −14.98, *p* < 0.001, *d* = 2.62. Thus, as predicted, as interactions between teachers and students became more blatantly racist, they were increasingly associated with higher perceptions of microaggressions.

**Table 1:** Descriptives and bivariate correlations of the main variables from Study 1 and Study 2.

	M	SD	1	2	3	4	5	6	7	
Study 1										
1. Gender^a^			—							
2. Migration background^b^			0.07	—						
3. Teaching experience^c^	27.83	37.24	−0.07	0.03	—					
4. Color‐evasiveness	3.46	1.22	0.11	−0.01	−0.06	—				
5. Exaggeration of cultural differences	4.10	1.02	0.00	0.06	0.08	−0.03	—			
6. Negation of positive emotions	4.89	1.30	−0.07	0.02	0.12	0.02	0.40**	—		
7. Implicit prejudice	−0.04	0.38	0.06	−0.05	0.03	−0.02	−0.11	−0.02	—	
Study 2										
1. Gender			—							
2. Migration background			0.09	—						
3. Teaching experience	26.52	53.65	0.05	−0.04	—					
4. Exaggeration of cultural differences	3.72	1.04	0.04	−0.01	0.11	—				
5. Negation of positive emotions	4.76	1.25	−0.19**	−0.08	0.14	0.32**	—			
6. Implicit prejudice	−0.09	0.40	−0.16*	−0.10	−0.04	0.04	−0.03	—		
7. Empathy	5.72	1.18	−0.04	0.07	0.05	−0.18*	0.08	0.03	—	
8. Critical reflection	5.95	1.13	−0.19*	−0.02	0.16*	0.08	0.16*	0.10	0.37***	—

*Note:* Study 1 (*N* = 147), Study 2 (*N* = 184).

^a^ 0 = female, 1 = male.

^b^ 0 = no, 1 = yes.

^c^ Teaching experience is expressed in the number of weeks.

* *p* < 0.05

** *p* < 0.01

*** *p* < 0.001.

H1b also received support. Ambiguity had a significant main effect on projected negative student sense of school belonging, *F*(2, 143) = 173.18, *p* < 0.001, *η*_p_^2^ = 0.71. In the school performance vignettes, participants projected significantly stronger negative student sense of school belonging in the low‐ambiguity condition (M = 5.42, SD = 1.27) than in the high‐ambiguity condition (M = 4.54, SD = 1.41), *t*(73) = −5.58, *p* < 0.001, *d* = 0.83. Both were significantly higher than the control condition (M = 2.17, SD = 1.30), all *ps* < 0.001. In the school normative behavior vignettes, the same pattern was observed: low‐ambiguity condition (M = 5.27, SD = 1.37) was higher than high‐ambiguity condition (M = 4.24, SD = 1.36), *t*(73) = −4.82, *p* < 0.001, *d* = 0.82, and control condition (M = 3.20, SD = 1.41), *t*(73) = −10.34, *p* < 0.001, *d* = 1.91. These findings indicate that more blatantly racist scenarios were associated with stronger negative students' sense of school belonging.

The regression model predicting perceived microaggressions and projected negative student sense of school belonging is reported in Tables 2 and 3. Partially in line with H2a, results showed that implicit prejudiced attitudes were negatively related to perceived microaggressions in the school‐performance scenario (*β* = −0.27, *p* = 0.041) but not in the normative situations. Contrary to what we expected, both subscales of subtle prejudice (i.e., the exaggeration of cultural differences and the negation of positive emotions) were positively related to perceived microaggressions in the normative scenario (*β* = 0.25, *p* < 0.05; *β* = 0.23, *p* < 0.05; respectively), indicating that participants with high levels of subtle prejudice seemed to be aware that the teacher in the vignette was biased.

**Table 2:** Hierarchical multiple regression analyses predicting perceived microaggressions in the school performance and school normative scenarios in Study 1.

Predictor	School performance			School normative		
	*B* [95% CI]	SE	*β*	*B* [95% CI]	SE	*β*
Constant	3.814***	0.60		1.513*	0.71	
Gender^a^	0.18 [−0.28; 0.64]	0.23	0.10	0.54 [0.07; 1.01]	0.24	0.26*
Migration background^b^	0.05 [−0.43; 0.53]	0.24	0.02	0.09 [−0.48; 0.65]	0.28	0.04
Teaching experience^c^	0.00 [−0.00; 0.00]	0.00	0.17	0.00 [−0.01; 0.01]	0.00	0.10
Color‐evasiveness	−0.03 [−0.17; 0.14]	0.08	−0.04	0.03 [−0.15; 0.22]	0.09	0.04
Exaggeration of cultural differences	0.09 [−0.12; 0.30]	0.11	0.12	0.25 [0.02; 0.48]	0.12	0.25*
Negation of positive emotions	−0.04 [−0.12; 0.30]	0.08	−0.07	0.19 [−0.00; 0.37]	0.09	0.23*
Implicit prejudice	−0.55 [−1.08; −0.02]	0.27	−0.27*	0.48 [−0.11; 1.06]	0.29	0.18

*Note: R* ^2^ = 0.12 for school performance. *R* ^2^ = 0.25 for school normative.

^a^ 0 = female, 1 = male.

^b^ 0 = no, 1 = yes.

^c^ Teaching experience is expressed in the number of weeks.

* *p* < 0.05

*** *p* < 0.001.

**Table 3:** Hierarchical multiple regression analyses predicting perceived students' negative sense of school belonging in the school performance and school normative scenarios in Study 1.

Predictor	School performance			School normative		
	*B* [95% CI]	SE	*β*	*B* [95% CI]	SE	*β*
Constant	2.507***	0.69		1.921**	0.66	
Gender^a^	0.34 [−0.19; 0.86]	0.26	0.17	0.40 [−0.04; 0.84]	0.22	0.21
Migration background^b^	−0.21 [−0.76; 0.34]	0.27	−0.10	0.32 [−0.20; 0.85]	0.26	0.14
Teaching experience^c^	0.00 [−0.00; 0.01]	0.00	0.10	0.00 [−0.01; 0.01]	0.00	0.01
Color‐evasiveness	0.11 [−0.07; 0.28]	0.09	0.16	−0.01 [−0.19; 0.16]	0.09	−0.01
Exaggeration of cultural differences	0.04 [−0.20; 0.28]	0.12	0.05	0.30 [0.08; 0.51]	0.11	0.33**
Negation of positive emotions	0.11 [−0.08; 0.29]	0.09	0.17	0.11 [−0.07; 0.28]	0.09	0.14
Implicit prejudice	−0.24 [−0.86; 0.37]	0.31	−0.11	0.15 [−0.40; 0.69]	0.27	0.05

*Note: R* ^2^ = 0.12 for school performance. *R* ^2^ = 0.24 for school normative.

^a^ 0 = female, 1 = male.

^b^ 0 = no, 1 = yes.

^c^ Teaching experience is expressed in the number of weeks.

** *p* < 0.01

*** *p* < 0.001.

Regarding the projected negative student sense of school belonging, only a positive association with exaggeration of cultural differences in the normative scenario was found (*β* = 0.33, *p* = 0.01), meaning that higher levels of one form of subtle prejudice were associated with projecting more negative consequences for the student depicted in the scenario. Color‐evasiveness and all control variables were not associated with perceived microaggressions neither nor students' negative sense of school belonging.

Relative to the set of scenarios in Study 1 (school‐related performance vs. school normative behavior), our participants were less likely to consider the teacher to be racist and the consequences on students less severe in the normative behavior vignettes. These differences in perceptions may be attributed to greater uncertainty in terms of intent and harm surrounding the microaggressions in the normative behavior. When assessing the associations between attitudinal dispositions and teacher noticing, we only focused on negative psychological characteristics (implicit and explicit prejudice) and neglected more positive ones. More specifically, we did not measure whether critical reflection and empathy could predict teacher noticing of microaggressions beyond implicit and explicit prejudice. We addressed both of these concerns in Study 2 with a larger sample, also to clarify the inconsistencies related to implicit prejudice. Implicit prejudice was only significantly associated with perceiving micro‐aggressions in the school performance scenario, but not in the normative scenario. There were no associations with projected negative sense of school belonging in either type of scenario. Study 2 thus served to ascertain the robustness of the association between implicit prejudice and the ability to notice microaggressions.

## 6 Study 2

### 6.1 Methods

#### 6.1.1 Participants

For Study 2, following our statistical power calculations (see preregistration) and due to limited study resources (each participant again received a 5‐euro gift card), a stopping rule of 200 participants was applied. The sample included 204 preservice teachers. Twenty participants were excluded because reported to have a Turkish heritage. Of the remaining 184 respondents (M_age_ = 23.2 years old, SD = 4.1; 64.7% females), most attended teacher training in the Ruhr area. About one fourth (24.5%) had an immigrant background. Participants were either enrolled in a primary (23.1%) or secondary teacher education program (76.9%).

#### 6.1.2 Measures, Study Design, and Procedure

SA‐IAT, two subscales of subtle prejudice (i.e., the exaggeration of cultural differences between groups *ω* = 0.70, and the denial of positive emotions, *r*_Spearman‐Brown_ = 0.58, *p* < 0.001), perceived microaggressions (*ω*_s_ = 0.77−0.88), and students' negative sense of school belonging (*ω*_s_ = 0.52−0.85) were measured using the same materials and items as in Study 1. For perceived microaggressions, we fit a confirmatory factor analysis model on the items with one factor per experimental condition. The fit of the three‐factor model was good, *χ* ^2^(24) = 34.687, CFI = 0.985, RMSEA = 0.047 [90% CI 0.00; 0.080], SRMR = 0.049. The standardized factor loadings for these items ranged from 0.48 to 0.86, signaling a good measurement of these three levels of perceived microaggressions. Similarly, for students' negative sense of school belonging, we ran confirmatory factor analysis on the items with one factor per experimental condition. The fit of the three‐factor model was good, *χ* ^2^(24) = 59.342, CFI = 0.918, RMSEA = 0.085 [90% CI 0.058; 0.113], and SRMR = 0.067. The standardized factor loadings for these items ranged from 0.40 to 0.97. There was only one exception, for one item of the scale in the control condition that reported a low factor loading (0.07). Given that the overall factor structure was supported, we retained this item for subsequent analyses. Only the set of vignettes depicting a school performance scenario used in Study 1 was retained in Study 2. For SA‐IAT, split‐half reliability, assessed through the odd‐even method, was adequate (*r* = 0.85).

Contrary to Study 1, we used a within‐subject design, in which all participants responded to one set of vignettes containing only the school performance scenario. But similarly to Study 1, participants first completed the SA‐IAT, then read each vignette, and subsequently responded to items on the perceived microaggression and students' negative sense of school belonging scales, concluding the experiment with demographics. The experiment was conducted online using Pavlovia and Unipark. Data were collected in January through May 2024.

##### 6.1.2.1 Critical Reflection

Adapted from Schwarzenthal et al. (2022), five items tapping into the critical reflection dimension of critical consciousness, namely awareness of systemic inequalities, were used (*ω* = 0.88, e.g., “People of different backgrounds do not always have the same opportunities in Germany”). The response scale ranged from 1 (*does not apply at all*) to 7 (*fully applies*), with higher scores indicating greater critical reflection. The fit of the one‐factor model was good *χ* ^2^(5) = 7.047, CFI = 0.995, RMSEA = 0.045 [90% CI 0.000; 0.114], and SRMR = 0.021. The standardized factor loadings for these items ranged from 0.62 to 0.88.

##### 6.1.2.2 Empathy

To measure empathy, two items tapping into empathic concern and two items tapping into perspective taking developed by Paulus (2009) were used (*ω* = 0.90, e.g., “I feel warm‐hearted feelings for people who are less fortunate than I am” and “Before I criticize someone, I try to imagine how I would feel in their place”). The response scale ranged from 1 (*does not apply at all*) to 7 (*fully applies*), with higher scores indicating greater of empathy. We ran a CFA on the items, and the modification indices indicated a correlated error between two items that both referred to emphatic concern. With this correlated error, the fit of the one‐factor model was good *χ* ^2^(1) = 0.148, CFI = 1.000, RMSEA = 0.000 [90% CI 0.000; 0.136], and SRMR = 0.002. The standardized factor loadings for these items ranged from 0.76 to 0.89.

#### 6.1.3 Analytic Strategy

To examine H1a and H1b, we conducted repeated measures, within factor ANOVA, with microaggression condition (i.e., control, high ambiguity, and low ambiguity) as the repeated measures factor, with each dependent variable separately (i.e., perceived microaggressions and projected negative student sense of school belonging), proceeded by checking the assumption of sphericity (Mauchly's test), and followed by planned comparisons across microaggression condition with Bonferroni correction. To test H2a and H2b, we ran separate multiple regression analysis regressing perceived microaggressions and student negative sense of school belonging on critical reflection, empathy, subtle prejudice toward Turkish origin individuals, and implicit prejudice, while controlling for participants' migration background, teaching experience, and gender. Similar to Study 1, perceived microaggressions and projected negative student sense of school belonging were calculated as a mean score for each participant across the microaggression ambiguity conditions. All analyses were conducted in SPSS and *R*.

### 6.2 Results

Table 1 shows descriptives and bivariate correlations for all study variables. Empathy and critical reflection were positively correlated (*r* = 0.37, *p *< 0.01), indicating that participants who reported high levels of empathy also reported high levels of critical reflection. Mauchly's test indicated that the assumption of sphericity held, *χ* ^2^(2) = 1.55, *p* = 0.462. The results showed that ambiguity had a significant main effect on perceived microaggressions, *F*(2, 366) = 603.72, *p* < 0.001, *η*_p_^2^ = 0.77, supporting H1a. Planned paired sample *t‐*tests indicated that the mean of perceived microaggressions of the low‐ambiguity condition (M = 6.20, SD = 1.14), was significantly higher than that of the high‐ambiguity condition (M = 3.93, SD = 1.73), *t*(183) = −17.08, *p* < 0.001, *d* = 1.89, which was significantly higher than the control condition (M = 1.71, SD = 1.10) *t*(183) = −16.98, *p* < 0.001, *d* = 1.85. Thus, as predicted and in line with the results in Study 1, as interactions between teachers and students became more blatantly racist, they were increasingly associated with higher perceptions of microaggressions.

Because Mauchly's test indicated that the assumption of sphericity had been violated, *χ* ^2^(2) = 15.16, *p* < 0.05, degrees of freedom were corrected using Huynh−Feldt estimate of sphericity (*ε* = 0.58) when reporting results for H1b. Ambiguity had a significant main effect on projected negative student sense of school belonging, *F*(2, 386.27) = 251.96, *p* < 0.001, *η*_p_^2^ = 0.58. This indicates that participants mean of student of the low‐ambiguity condition (M = 5.71, SD = 1.12) was significantly higher than that of the high‐ambiguity condition (M = 4.53, SD = 1.52), *t*(183) = 10.13, *p* < 0.001, *d* = 1.04, which in turn was significantly higher than the control condition (M = 2.86, SD = 1.51), *t*(183) = −13.13, *p* < 0.001, *d* = 1.54. Thus, Study 2 provided further evidence of preservice teachers' ability to attend and interpret ethnic microaggressions.

The multiple regression models predicting perceived microaggressions and projected negative student sense of school belonging are reported in Table 4. Preservice teachers' degree of critical reflection positively predicted perceived microaggressions (*β* = 0.30, *p* < 0.001), indicating that those with higher levels of critical consciousness reported higher levels of awareness to microaggressions in the vignettes. For projected negative student sense of school belonging, the model accounted for 16% of variance. Similarly, for the first dependent variable, participants' degree of critical reflection positively projected a negative student sense of school belonging (*β* = 0.32, *p* < 0.001). Contrary to what predicted, empathy was not related to teacher noticing.^2^ Unlike Study 1, implicit prejudice was not related to attending nor interpreting microaggressions. All control variables were not significantly associated with the dependent variables, except for migration background, which was negatively associated with perceived microaggressions (see Table 4). This finding indicates that preservice teaching students without a migration background were more likely to perceive microaggressions throughout the scenarios than students with a migration background.

**Table 4:** Hierarchical multiple regression analyses predicting perceived microaggressions and perceived students' negative sense of school belonging in Study 2.

Predictor	Perceived microaggressions			Perceived students' negative sense of school belonging		
	*B* [95% CI]	SE	*β*	*B* [95% CI]	SE	*β*
Constant	3.026***	0.60		2.494***	0.70	
Gender^a^	−0.20 [−0.46; 0.06]	0.13	−0.11	−0.10 [−0.40; 0.19]	0.15	−0.05
Migration background^b^	−0.40 [−0.73; −0.07]	0.17	−0.17*	−0.11 [−0.49; 0.27]	0.19	−0.04
Teaching experience^c^	0.00 [−0.00; 0.00]	0.00	0.03	−0.00 [−0.00; 0.00]	0.00	−0.05
Exaggeration of cultural differences	−0.02 [−0.15; 0.11]	0.07	−0.02	0.11 [−0.04; 26]	0.08	0.11
Negation of positive emotions	0.06 [−0.05; 0.16]	0.05	0.08	0.06 [−0.06; 18]	0.06	0.07
Implicit prejudice	−0.20 [−0.50; 0.11]	0.15	−0.10	−0.00 [−0.36; 0.34]	0.18	−0.00
Empathy	0.04 [−0.08; 0.16]	0.06	0.06	−0.04 [−0.18; 0.10]	0.07	−0.04
Critical reflection	0.25 [0.13; 0.37]	0.06	0.30***	0.30 [0.16; 0.44]	0.07	0.32***

*Note: R* ^2^ = 0.19 for perceived microaggression, *R* ^2^ = 0.16 for perceived students' negative sense of school belonging.

^a^ 0 = female, 1 = male.

^b^ 0 = no, 1 = yes.

^c^ Teaching experience is expressed in the number of weeks.

* *p* < 0.05

*** *p* < 0.001.

## 7 Discussion

Using a vignette experiment, this was the first psychological investigation of how preservice teachers noticed subtle forms of racism in the school ecology, known as microaggressions. The use of an experimental design and written vignettes facilitated an examination of two related processes of racial noticing, namely attending and interpreting, across two samples of preservice teachers in Germany. Additionally, the current study explored whether participants' attitudinal dispositions in the forms of implicit and explicit prejudice, as well as critical reflection and empathy, can account for these differences in noticing microaggressions. Overall, across the two studies, our results indicated noticing ability in detecting microaggressions directed toward Turkish heritage students when deliverers are teachers. However, we found less consistent evidence in the two samples for the role of participants' attitudinal dispositions as predictors of racial noticing.

Results suggest that participants perceived greater microaggression against Turkish heritage students as the ambiguity of discrimination decreased. Furthermore, preservice teachers from the two studies expected student targets to experience a lower sense of school belonging following more blatant microaggressions. Thus, teachers in training can detect nuances in the ambiguous nature of ethnic microaggressions directed at students in observed interactions with a teacher and interpret them by predicting a more negative impact among students targeted by more explicit racism. These findings showed medium‐to‐large effect sizes, giving confidence in the results. These results are also consistent with prior vignette studies from the United States focusing on third‐party observations of racial and ethnic microaggressions (Tao et al. 2017; Zou and Dickter 2013) as well as gender microaggressions (Basford et al. 2014; Offermann et al. 2013). Importantly, our findings extend these observations to other contexts (i.e., schools) and groups (i.e., preservice teachers). Importantly, while previous qualitative studies have suggested that teachers often struggle to recognize and address microaggressions (Burleigh and Wilson 2021; Pearce 2019), our results indicate that preservice teachers are, in fact, sensitive to variations in the ambiguity of microaggressions and can anticipate their potential impact on students. Contrary to prior qualitative work (Burleigh and Wilson 2021; Pearce 2019), our data show that teachers in training may be more attuned to microaggressions than previously assumed—at least in experimental settings where contextual cues highlight discriminatory behavior. Preservice teachers are encouraged not to remain passive bystanders but rather to actively respond to discriminatory actions in educational settings. While it is premature to conclude that teacher noticing was enhanced during teaching education, it is possible that the debates on racism following the brutal killing of George Floyd and the Black Lives Matter protests can have also an influence on the awareness of racism at the time of the survey, as it is shown in other nationally representative samples in Germany (DeZIM [Deutsches Zentrum für Integrations‐ und Migrationsforschung] 2022).

### 7.1 The Role of Psychological Characteristics

In Study 1, the pattern of the results was only partially consistent with our hypotheses that implicit prejudice would predict both attending and interpreting microaggressions. Specifically, we found that high levels of implicit prejudice were negatively related to perceived greater microaggression (attending) but only in the school‐performance scenario, and implicit prejudice was not associated to interpreting in neither scenario. In Study 2, we were unable to replicate the findings for a predictive relationship between implicit prejudice and perceived microaggressions and projected negative student sense of school belonging. Since the vignettes depicted microaggressions in a verbal form (e.g., racist comments by a teacher), it could be argued that individuals with higher implicit prejudice may struggle to recognize and interpret subtler, nonverbal forms of microaggressions, such as avoiding the seat next to a person with a marginalized identity. While these nonverbal forms of bias generally operate below the level of awareness and are difficult for well‐intended individuals to control, racist comments, differing in ambiguity, may be interpreted as intentional and thus are more readily perceived as a form of overt discrimination, also for individuals who report high levels of implicit prejudice (Dovidio et al. 2018).

Unexpectedly, we found that subtle prejudice, especially the exaggerations of cultural differences, was positively linked to perceived greater microaggression (attending) and perceptions of students' negative sense of school belonging (interpreting) in Study 1 (but not in Study 2). One explanation may be that, despite individuals who harbor higher levels of subtle prejudice may have a strong in‐ and out‐group reference point (Pettigrew and Meertens 1995), they still perceive ethnic microaggressions as discriminatory to a certain degree. Still, our findings contrast with previous research showing a positive association between subtle prejudice with discriminatory behaviors (Meertens and Pettigrew 1997). This discrepancy raises the question of whether this measure, in particular the exaggeration of the cultural differences dimension, may really be indicative of explicitly underlying hostility and negative feelings toward minoritized groups of students or if it primarily reflects perceptions of social reality that acknowledge differences between majority and minoritized groups (Coenders et al. 2001). Notably, although the means of exaggeration of cultural differences between groups, and the denial of positive emotions across the two samples were above the scale midpoint, this scale is somewhat outdated, and these items may not be perceived so subtle, a finding also highlighted in a study conducted recently in Germany by Stanke et al. (2024) that administered this scale to participants who were potential targets of ethnic‐racial prejudice.

For color‐evasiveness, we found no significant association with the ability to detect microaggressions in Study 1. Mean values of ignoring differences were below the mid‐point of the scale but still higher (about 1 SD) than in previous studies (Civitillo et al. 2021). Color‐evasiveness was also not positively associated with the two dimensions of subtle prejudice. One explanation for the nonsignificant association between color‐evasiveness and the ability to detect micro‐aggressions could be that, unlike the subtle prejudice scales, color‐evasiveness directly taps into how teachers should treat students, and therefore, carry‐over effects of measuring color‐evasiveness after reading the vignettes cannot be ruled out.

For empathy, we found no association with teacher noticing, which was unexpected. Empathy has well‐known negative associations with prejudice (Bäckström and Björklund 2007; Pedersen et al. 2004) and was also negatively associated with the exaggeration of group differences dimension of subtle prejudice in our study. Yet, there are also studies showing that empathy is not always beneficial, particularly in real‐world intergroup interactions, because individuals tend to show ingroup bias in empathy—that is, they are more likely to empathize with ingroup rather than with outgroup members (Cikara et al. 2014). It is important to keep in mind that we measured general empathy and not ethnocultural empathy (i.e., empathy with ethnically minoritized groups). Moreover, in contrast with prior research showing that affective and cognitive empathy can have distinct associations with ethnic prejudice (Bobba and Crocetti 2022) and intergroup behaviors (Bayram Özdemir et al. 2020), our robustness checks separating affective (empathic concern) and cognitive (perspective taking) empathy confirmed the absence of significant associations with teacher noticing. Finally, the relatively low emotional vividness of the vignettes could also have constrained the role of empathy in shaping teachers' noticing.

But the findings for critical reflection are encouraging. Critical reflection reflects a heightened awareness of the world and the power structures that shape it. Ethnic microaggressions, especially when these exchanges occur between majority teachers and minoritized students, represent a concrete situation of imbalanced power. Unlike solely empathizing with the target of discrimination, critical reflection may represent a step forward and thus translate to truly attending and carefully interpreting these exchanges as racist (Fu et al. 2024; Steketee et al. 2021). Among adolescents, experiences of marginalization and oppression can help enhance the development of critical reflection (Diemer et al. 2021). For members of socially dominant groups like teachers, we urge the need to examine interventions promoting teachers' development of critical reflection. Facilitating the Identity Project, an 8‐week classroom‐based intervention (see Pevec‐Zimmer et al. 2024 and Ulbricht et al. 2024 for empirical evidence in Germany), may represent a way of raising awareness of discrimination against these students and thus help with one domain of critical consciousness, namely critical reflection of oppressive systems.

### 7.2 Limitations and Future Research Directions

This study has some important limitations that should be highlighted. First, the present investigation did not focus on *responding*, which is the third process of teacher racial noticing and reflects the behavioral component of teacher noticing. Next to attending and interpreting, future research could use classroom observations or Virtual Reality to explore how all these processes are interrelated. While an experimental design offers control over specific variables (e.g., manipulation of discrimination) and this controlled design allows for isolation of specific effects, it may limit the generalizability of findings to real‐world classroom settings. Thus, future studies should examine teacher noticing in more naturalistic contexts to enhance ecological validity. Second, we focused on a single dimension of oppression based on ethnicity and targeting a specific group of students (i.e., Turkish heritage male students), suggesting that studies should consider replicating our analyses investigating the multiple identity dimensions of oppression and other stigmatized groups (e.g., refugee students). Third, our samples were rather homogenous samples of preservice teachers, although both samples reflected most teaching population in Germany. Relatedly, the homogenous nature of the present samples offers a conservative assessment of our proposed hypotheses, suggesting that future studies may find larger effect sizes among more heterogeneous samples, including larger groups of teachers from minoritized backgrounds. Fourth, some reliabilities for the scales measuring the dependent variables measured across three conditions (control, low, and high ambiguity) were suboptimal. Several factors may explain this, including that internal consistency is typically used for trait‐like concepts and may not fully reflect the reliability of context‐dependent measures like ours. Future studies could further help developing more reliable instruments for experimental studies on microaggressions. In addition, future research should include multiple vignettes per microaggression condition. This would allow researchers to examine interindividual differences in the ability to detect microaggressions under different conditions (e.g., at different levels of ambiguity).

### 7.3 Implications and Conclusion

There is a need to find ways of talking about racism during teacher training more openly in the European context. How to address it can be challenging as other qualitative work has shown (e.g., Burleigh and Wilson 2021). Although this study made use of an experimental design, the methodology we implied (short classroom scenarios) can be used to discuss racism in initial teacher education classes. In fact, we have done so with great effect in our own classes and find the discussion of the highly ambiguous scenarios particularly instructive because of the highly varied nature of responses from students. It may foster the conversation to demonstrate in which ways racism can manifest in the school context and can be initiated by other students as well as by teachers and other school staff. It is important to stress to preservice teachers that racism is not only overtly expressed but can take different subtle forms and reasoning around the cumulative experiences of such actions. In addition, it is equally important to engage in discussion with teachers in training about how such incidents are not merely interpersonal interactions, but rather symptomatic of broader systemic inequities and historical legacies of oppression (Syed 2021).

Research on microaggressions among teachers remains limited. Although these forms of racism may be subtle, the consequences are not trivial, especially for young students in a supposedly safe space like schools. Future research, practice, and policy can thus benefit from understanding more about the relationships between teacher noticing and microaggressions and pursuing new insights on the role of teacher psychology.

## Supporting information

Study_1_Supplementary_Materials.

Study_2_Supplementary_Materials.

## Data Availability

The data that support the findings of this study are available on request from the corresponding author. The data are not publicly available due to privacy or ethical restrictions. The data that support the findings of this study are openly available in OSF at https://osf.io/r7swm/.

## Endnotes

1 Cohen's *d* for the paired sample *t*‐tests were calculated using the means, standard deviations, and the correlations between the two variables tested.

2 We ran additionally sensitive analyses to test the robustness of this finding. When omitting critical reflection, empathy remained unrelated to teacher noticing. Conversely, when excluding empathy, critical reflection remained significant and in the expected direction. In another set of analyses, we separated the two facets of empathy (i.e., two items for empathic concern and two items for perspective taking), but the results remain nonsignificant.
