# Supplementary material for: Investigating Preservice Teachers' Competence to Notice Ethnic Microaggressions in the Classroom
Source: J Community Psychol. 2025 Nov 19;53(8):e70056. doi: 10.1002/jcop.70056 (PMC12629893; doi:10.1002/jcop.70056)
Supplement: Supplementary file 2 — Study_2_Supplementary_Materials. [file JCOP-53-0-s002.docx]

**Title Project**: Investigating pre-service teachers’ competence to notice ethnic microaggressions in the classroom (Study 2)

Single Attribute Implicit Association Test (SA-IAT) (Greenwald et al., 1998; Penke et al., 2006):

**Original version in German**: Beschreibungen - Charaktereigenschaft

religiös

traditionell

frauenfeindlich

segregiert

unangepasst

intolerant

**English translation**: Descriptions - Character trait

religious

traditional

misogynistic

segregated

non-conformist

intolerant

SCENARIO - School-related performance

**CONDITION: HIGH AMBIGUITY**

**Original version in German**: *Nachfolgend präsentieren wir Ihnen die ingesamt drei Beschreibungen von Situationen in der Schule. Bitte lesen Sie diese aufmerksam durch. Danach gelangen Sie jeweils mit der Leertaste weiter, um einige Fragen dazu zu beantworten.*

1. Es ist der erste Schultag der 5. Klasse an einer Gesamtschule. Die Lehrerin Frau Haas bittet die Schüler etwas über sich zu erzählen. Emre, ein Schüler mit türkischen Wurzeln, sitz in der ersten Reihe und wird von Frau Haas angesprochen.

*Frau Haas: Emre, was sind deine Hobbies?*

*Emre: Entschuldigung?*

*Frau Haas: Was sind deine Hobbies? Hast du mich nicht verstanden?*

**English translation**: *Below we present a total of three descriptions of situations at school. Please read them carefully. Then press the space bar to answer a few questions.*

1. It is the first day of school for year 5 at a comprehensive school. The teacher, Ms. Haas, asks the pupils to tell her something about themselves. Emre, a pupil with Turkish roots, is sitting in the front row and is approached by Ms. Haas.

*Ms. Haas: Emre, what are your hobbies?*

*Emre: Excuse me?*

*Ms. Haas: What are your hobbies? Did you not understand me?*

**CONDITION: LOW AMBIGUITY**

**Original version in German**: Die Klasse 8b kriegt ihre erste Klassenarbeit bei ihrem neuen Deutschlehrer, Herrn Jäger, zurück. Herr Jäger übergibt den Schülern einzeln ihre Klassenarbeiten. Kerem, ein Schüler mit türkischen Wurzeln, bekommt seine Arbeit.

*Herr Jäger: Betül, hier ist deine Arbeit.*

*Kerem: Toll! Eine 2+!*

*Herr Jäger: Du warst wirklich gut für einen türkischen Schüler!*

**English translation**: Class 8b gets their first test back from their new German teacher, Mr. Jäger. gives the pupils their class work individually. Kerem, a pupil with Turkish roots, gets his work.

*Mr. Jäger: Betül, here is your work.*

*Kerem: Great! A 2+.*

*Mr. Jäger: You did really well for a Turkish pupil!*

**CONDITION: CONTROL**

**Original version in German**: In der 12. Klasse muss bald eine Facharbeit in einem bestimmten Fach geschrieben werden. Serkan, ein Schüler mit türkischen Wurzeln, ist sich noch unsicher, worüber er seine Facharbeit schreiben soll. Also geht er zu seinem Englischlehrer, Herrn Schmidt, und fragt ihn nach seinem Rat.

*Serkan: Ich würde meine Facharbeit gerne über etwas schreiben, was mich wirklich interessiert.*

*Herr Schmidt: Das ist die richtige Einstellung! Was interessiert dich denn aktuell?*

EN: In the 12th grade, a term paper has to be written soon in a particular subject. Serkan, a student with Turkish roots, is still unsure about he should write his paper on. He goes to his English teacher, Mr. Schmidt, and asks for his advice.

*Serkan: I would like to write my paper about something that really interests me.*

*Mr. Schmidt: That’s the right attitude! What are you currently interested in?*

**Perceived microaggression (Adapted from previous research on microaggressions; Basford et al., 2014; Tao et al., 2017)**

**Original version in German**: *Bitte bewerten Sie die folgenden Aussagen indem Sie den Grad Ihrer Zustimmung dazu mit Hilfe einer Skala von 1 (“trifft überhaupt nicht zu”) bis 7 (“trifft völlig zu”) angeben.*

1. Der Lehrer war unsensibel gegenüber der kulturellen Herkunft des Schülers.
2. Der Lehrer schien sich der verschiedenen Erscheinungsformen von Rassismus nicht bewusst zu sein.
3. Der Lehrer schien gegenüber verschiedenen Kulturen voreingenommen zu sein.

EN: *Please rate the following statements by indicating your level of agreement using a scale from 1 (“strongly disagree”) to 7 (“strongly agree”).*

1. The teacher was insensitive to the student's cultural background.
2. The teacher seemed unaware of the different manifestations of racism.
3. The teacher seemed to be prejudiced against different cultures.

**Negative perceived student sense of school belonging (Adapted from previous research on microaggressions; Offermann et al., 2013)**

**Original version in German**: *Bitte bewerten Sie die folgenden Aussagen indem Sie den Grad Ihrer Zustimmung dazu mit Hilfe einer Skala von 1 (“trifft überhaupt nicht zu”) bis 7 (“trifft völlig zu”) angeben.*

1. Die Antwort der Lehrkraft hat einen Einfluss darauf, wie zugehörig sich (Name des Schülers) zur Klasse fühlt.

2. Die Antwort der Lehrkraft führt dazu, dass (Name des Schülers) sich weniger zugehörig zur Klasse fühlt.

3. Die Antwort der Lehrkraft führt dazu, dass (Names des Schülers) weniger motiviert ist, etwas für die Schule zu tun.

**English translation**: *Please rate the following statements by indicating your level of agreement using a scale from 1 (“strongly disagree”) to 7 (“strongly agree”).*

1. The teacher's answer has an influence on how much (student's name) feels part of the class.
2. The teacher's answer makes (student's name) feel less like a member of the class.
3. The teacher's answer makes (student's name) feel less motivated to do something for school.

**Empathy**

**Original version in German**: *Sie werden jetzt eine Reihe von Aussagen lesen, die jeweils bestimmte (verallgemeinerte) menschliche Eigenschaften oder Reaktionen beschreiben, die alle etwas mit Gefühlen zu tun haben.*

#### *Bitte bewerten Sie die folgenden Aussagen indem Sie den Grad Ihrer Zustimmung dazu mit Hilfe einer Skala von 1 (“trifft auf mich nicht zu”) bis 7 (“trifft auf mich zu”) angeben.*

#### 1. Ich empfinde warmherzige Gefühle für Leute, denen es weniger gut geht als mir.

#### 2. Mich berühren Dinge sehr, auch wenn ich sie nur beobachte.

#### 3. Wenn mir das Verhalten eines anderen komisch vorkommt, versuche ich mich für eine Weile in seine Lage zu versetzen.

#### 4. Bevor ich jemanden kritisiere, versuche ich mir vorzustellen, wie ich mich an seiner Stelle fühlen würde.

#### English translation: *You will now read a series of statements, each of which describes certain (generalized) human characteristics or reactions, all of which have something to do with feelings. Please rate the following statements by indicating your level of agreement using a scale from 1 (“does not apply to me”) to 7 (“applies to me”).*

#### I feel warm-hearted feelings for people who are less fortunate than I am.

#### Things touch me deeply, even if I only observe them.

#### If someone else’s behaviour seems strange to me, I try to put myself in their position for a while.

#### Before I criticize someone, I try to imagine what the situation looks like from their point of view.

#### Critical Consciouness

#### Original version in German: *Bitte bewerten Sie die folgenden Aussagen indem Sie den Grad Ihrer Zustimmung dazu mit Hilfe einer Skala von 1 (“trifft überhaupt nicht zu”) bis 7 (“trifft völlig zu”) angeben.*

#### 1. Menschen unterschiedlicher Herkunft nicht immer die gleichen Chancen in Deutschland haben.

#### 2. Menschen in Deutschland fremdenfeindliche Ansichten haben.

#### 3. Das deutsche Schulsystem nicht für alle Schüler die gleichen Möglichkeiten bietet.

#### 4. Menschen unterschiedlicher Herkunft in Deutschland oft unterschiedlich behandelt werden.

#### 5. Menschen bestimmter Herkunft manchmal mehr leisten müssen, um eine gute Arbeit zu bekommen.

#### English translation: *Please rate the following statements by indicating your level of agreement, using a scale from 1 (“strongly disagree”) to 7 (“strongly agree”).*

#### People from different backgrounds do not always have the same opportunities in Germany.

#### People in Germany hold xenophobic views.

#### The German school system does not offer equal opportunities for all students.

#### People of different origins are often treated differently in Germany.

#### People of certain backgrounds sometimes have to work harder to get a good job.

#### Subtle Prejudice Towards Turkish Origin Individuals (Ganter, 2001)

#### Original version in German: *In der folgenden Liste sind einige Dinge aufgeführt, die viele Leute als wichtige Aspekte von Gemeinsamkeiten oder Unterschieden zwischen verschiedenen Volksgruppen ansehen. Bitte bewerten Sie die folgenden Aussagen indem Sie den Grad Ihrer Zustimmung dazu mit Hilfe einer Skala von 1 (“sehr große Gemeinsamkeiten”) bis 7 (“sehr große Unterschiede”) angeben.*

#### 1. [Gemeinsamkeiten und Unterschiede zwischen hier lebenden Türken und Deutschen in Bezug auf] die Werte, zu denen die Kinder erzogen werden.

#### 2. [Gemeinsamkeiten und Unterschiede zwischen hier lebenden Türken und Deutschen in Bezug auf] die religiösen Überzeugungen und Praktiken.

#### 3. [Gemeinsamkeiten und Unterschiede zwischen hier lebenden Türken und Deutschen in Bezug auf] die sexuelle Moral oder das sexuelle Verhalten.

#### 4. Wie oft haben Sie Sympathie für die hier lebenden Türken empfunden?

#### Von 1 (sehr oft) bis 7 (sehr selten)

#### 5. Wie oft haben Sie Bewunderung für sie empfunden?

#### Von 1 (sehr oft) bis 7 (sehr selten)

#### English translation: *The following is a list of things that many people consider to be important aspects of similarities or differences between different ethnic groups. Please rate the following statements by indicating your level of agreement using a scale from 1 (“very much in common”) to 7 (“very different”).*

#### [Similarities and differences between Turks and Germans living here with regard to] the values to which the children are brought up.

#### [Similarities and differences between Turks and Germans living here with regard to] religious beliefs and practices.

#### [Similarities and differences between Turks and Germans living here in terms of] sexual morality or behavior.

#### How often have you felt sympathy for Turks living here?

#### *From 1 (very often) to 7 (very rarely).*

#### How often did you feel admiration for them?

#### *From 1 (very often) to 7 (very rarely).*

CONTROLS

**Original version in German**: Welches ist Ihr Geschlecht?

**English translation**: What is your gender?

**Original version in German**: Wieviel Praxiserfahrung haben Sie insgesamt in Wochen?

**English translation**: How much practical experience do you have in weeks?

**Original version in German**: Welche Schulform studieren Sie? “Grundschule”, “Haupt-, Real-, Gesamtschule”, “Gymnasium”, “Berufskolleg”)

**English translation**: What type of school are you studying? Primary school’, “Hauptschule”, “Realschule, Gesamtschule”, “Gymnasium”, “Berufskolleg”)

**Original version in German**: Haben Sie einen Migrationshintergrund? wenn ja, welchen?

**English translation**: Do you have a migration background?

**Original version in German**: Haben Ihre Eltern einen Migrationshintergrund?

**English translation**: Do your parents have a migration background?

**Original version in German**: Würden Sie sich selbst als Migrant_in bzw. Person mit Migrationshintergrund bezeichnen?

**English translation**: Would you describe yourself as a migrant or a person with a migration background?
